# Growth traits of crossbreds of Ankole with Brown Swiss, Holstein Friesian, Jersey, and Sahiwal cattle in Rwanda

**DOI:** 10.1007/s11250-017-1501-7

**Published:** 2017-12-19

**Authors:** Maximillian Manzi, Lotta Rydhmer, Martin Ntawubizi, Callixte Karege, Erling Strandberg

**Affiliations:** 1 Swedish University of Agricultural Sciences (SLU), Uppsala, Sweden; 2 Rwanda Agricultural Board (RAB), Kigali, Rwanda; 3 University of Rwanda (UR), Butare, Rwanda

**Keywords:** Breeds, Live weight, Growth traits, Crossbreeding

## Abstract

The objective of the study was to compare body weights and growth from birth to 18 months of age of various groups of crossbred cattle born from 1999 to 2007, being crossbreds of Ankole (A) with Brown Swiss (B), Holstein Friesian (F), Jersey (J), and Sahiwal (S). Average weights were 26.5 kg at birth, 161 kg at weaning, and 226 kg at 18 months. Both season and sex significantly affected birth weight (BW), weight at 8 and 18 months (W8 and W18), and average daily gain from weaning to 18 months (ADG18) and, unlike season, sex significantly affected average daily gain to 8 months and weaning age. The general trend was that average daily gain attained a maximum before weaning and thereafter decreased until 18 months. Least square means for AB and AF calves were comparable and significantly differed only for W18 and ADG18. AJ had the lowest BW but was comparable with AS, AJxS, and ASxJ for W8, age-adjusted weaning weight, and W18. Generally, AF was heavier than other breed groups, but the difference was smaller than expected probably because environmental conditions did not allow full expression of genetic potential for growth.

## Introduction

The Ankole is a medium-sized breed with males maturing at a live weight of 350 to 400 kg and females at 200 to 350 kg. Milk production per animal per day ranges between 1 to 6 kg (Kugonza et al. 2011). Newborn calves weigh about 14–23 kg and remain small for several months. The breed like other breeds in tropics is characterized by low-productivity and low-growth rates but highly adapted to harsh environments (Wurzinger et al. 2014). In contrast, *Bos taurus* breeds that are predominantly found in temperate countries have a high-production potential, but poor adaptation to tropical hash environment (Roschinsky et al. 2015). Therefore, crossbreeding of *B. indicus* with *B. taurus* breeds has been widely used in most African countries, including Rwanda, to combine the high-production potential of exotic breeds with the adaptability of the indigenous breeds.

Pre-weaning and post-weaning growth are important traits to select for in cattle (Pravia et al. 2014), but in Rwanda, there is a paucity of information on body weights and gains of Ankole cattle crossbreds. Therefore, information on pre-weaning and post-weaning growth was collected and analyzed to shed some light on comparative growth potential of different crossbred groups at a research station. The objective of this study was to identify suitable crosses by comparing body weights and weight gains of crossbreds of Ankole with Brown Swiss, Holstein Friesian, Jersey, and Sahiwal.

## Material and methods

### Location and climatic conditions

Data were from Songa Research Station (2^o^ 25’S, 29^o^ 48′E), located in the mid-altitude zone (1471 m.a.s.l), with short rains (season SRS) falling between September and December and long rains (season LRS) extending from March through May. The dry seasons extend between June and August (LDS) and January to February (SDS). The mean annual rainfall was 1087 mm and average temperature of 20.1 °C, with relative humidity of 77% (Mutimura et al. 2016). The natural grass is predominantly composed of *Brachiaria decumbens* and limited planted multipurpose trees like Calliandra calothyrsus and *Leucaena leucocephala*.

### Animal and herd management

The animals used in the study included 811 calves at birth, 781 calves at 8 months, 493 calves weaned, and 457 animals at 18 months of age. Female calves were mainly kept as potential replacement cows. Most male calves were sold for slaughter after 6 months, while others were sold to farmers as breeding bulls. Weaning was done in groups when calves were 8–13 months based on judgment of calf vigor and survivability. Partial milking was practiced, whereby calves suckled briefly to stimulate milk let-down and after milking were allowed to suckle residual milk. The animals were kept and grazed on paddocked natural pasture without supplementary feeds, and only calves were kraaled. Routine veterinary attention was provided to each animal.

### Data and statistical analysis

Data used in this study were obtained from crossbred calves born from 1999 to 2007 of Ankole (A) or crossbred dams (AJ and AS) with Brown Swiss (B), Friesian (F), Jersey (J), and Sahiwal (S) sires (semen). Breed groups (crosses) are designated by the combination of breed acronyms, e.g., AJ × S for an Ankole × Jersey crossbred mother with a Sahiwal father. For each calf, records included breed group, sex, dates, and weights from birth to 18 months of age. Weight records were taken monthly using weight balance, but for this study, the following weight traits were analyzed: (a) birth weight (BW) recorded within 24 h of birth, (b) weight at approximately 8 months (W8), (c) weaning weight (WWadj) adjusted to 10 months, (d) weaning weight unadjusted (WW), and (e) weight around puberty recorded at 18 months (W18). Furthermore, we analyzed the following growth rate traits: (a) average daily gain to 8 months (ADG8), computed as (W8-BW)/AGE8, (b) pre-weaning average daily gain (ADGW, from birth to weaning) computed as (WW-BW)/AGEW, (c) post-weaning average daily gain (ADG18, from weaning to 18 months) computed as (W18-WW)/(AGE18-AGEW), where AGE8 (AGE18) is the age of the animal at the monthly weighing closest to 8 (18) months of age and AGEW is the recorded age at weaning. Furthermore, we investigated the trait age at weaning (AGEW).

After removing observations with missing records, there were about 800 records for BW, W8, and ADG8 and around 400–500 records for the other traits (Table 1). The distribution of observations over sex and breed groups is shown in Table 2.

**Table 1:** The total number of observations, overall mean, SD, and range for all traits investigated

Trait^1^	Measure				Factor				
	*N*	Mean	SD	Range	bg^2^	Sex	Year	Season	bg × sex
BW	811	26.5	3.7	12–50	0.0001	0.0420	0.0001	0.0341	n.s.
W8	781	123	21.4	65–229	0.0004	0.0017	< 0.0001	0.0287	n.s.
WW	493	161	33.2	74–285	0.002	n.s.	< 0.0001	n.s	−^3^
W18	457	226	34.2	88–346	0.001	n.s.	< 0.0001	0.0133	−^3^
ADG8	779	0.40	0.09	0.16–0.65	0.0152	0.0067	0.0018	0.0004	n.s.
ADGW	485	0.41	0.09	0.17–0.80	0.0018	0.0057	< 0.0001	n.s	n.s
ADG18	424	0.36	0.10	0.07–0.74	0.0014	n.s	< 0.0001	n.s	−^3^
AGEW	491	330	67.4	125–583	0.1602	< 0.0001	0.0165	< 0.0001	0.0205

The levels of significance (*p* values) for all main effects and their possible interactions. Significance levels are given for the model where all significant effects are included and non-significant effects excluded

^1^ *BW* birth weight (kg), *W8* weight (kg) at 8 months of age, *WW* weaning weight (kg), *W18* weight (kg) at 18 months, *ADG8* average daily gain (g) to 8 months, *ADGW* pre-weaning daily gain (g), *ADG18* post-weaning daily gain (g) up to 18 months, *AGEW* age at weaning (days)

^2^ *bg* breed group

^3^Indicates that this interaction was not tested because one or both of the main effects were non-significant

**Table 2:** Distribution of observations over breed groups and sex classes

Trait^1^	Breed group^2^						Sex		
	AB	AF	AJ	AS	AJxS	ASxJ	Female	Male	Unknown
BW	41	46	346	189	86	103	338	170	303
W8	38	47	330	183	82	101	325	168	288
WW	39	38	162	128	61	65	320	160	13
W18	16	27	146	128	65	75	250	66	141
ADG8	38	46	330	182	82	101	325	168	286
ADGW	38	38	161	124	61	63	313	159	13
ADG18	13	27	129	121	61	73	237	64	123
AGEW	38	38	162	126	61	66	316	161	14

^1^ *BW* birth weight (kg), *W8* weight (kg) at 8 months of age, *WW* weaning weight (kg), *W18* weight (kg) at 18 months, *ADG8* average daily gain (g) to 8 months, *ADGW* pre-weaning daily gain (g), *ADG18* post-weaning daily gain (g) up to 18 months, *AGEW* age at weaning (days)

^2^ *AB* Ankole × Brown Swiss, *AF* Ankole × Holstein Friesian, *AJ* Ankole × Jersey, *AS* Ankole × Sahiwal, *AJxS* AJ × Sahiwal, *ASxJ* AS × Jersey

### Statistical analysis

Data were analyzed using General Linear Models (GLM) procedure of SAS (2012). The most complete model tested was as follows:

$$ {y}_{\mathrm{ijklm}}=\upmu +{\mathrm{bg}}_i+{\mathrm{sex}}_j+{\mathrm{yr}}_k+{\mathrm{seas}}_l+{\left(\mathrm{bg}\times \mathrm{sex}\right)}_{ij}+{\left(\mathrm{bg}\times \mathrm{seas}\right)}_{il}+{\left(\mathrm{sex}\times \mathrm{seas}\right)}_{jl}+{e}_{\mathrm{ijklm}} $$

where y_*ijklm*_ is the observation for a given trait; μ is the overall mean; bg_*i*_ is the fixed effect of breed group *i* (*i* = AB, AF, AJ, AS, AJxS, ASxJ); sex_*j*_ is the fixed effect of sex *j* (*j* = male, female, unknown); yr_*k*_ is the fixed effect of birth year *k* (*k =* 1999,…2007); seas_*l*_ is the fixed effect of birth season *l* (*l* = 1, 2, 3, 4); and *e*_*ijklm*_ is the random residual ~ ND (0, ${\sigma}_e^2$). For the weights at W8, WWadj, and W18, a linear regression on the actual age as a deviation from the expected age (240, 300, and 540 days) was included. Two-way interactions are shown within parentheses. Least Square Means (LSM) were computed to estimate differences among means of traits for different factors.

## Results

The number of observations, overall means, standard deviations, and ranges observed for all traits studied together with significance levels for factors in the model and the least square means for the all main effects are presented in Table 1. The distribution of observations over breed groups and sex classes is shown in Table 2. Least Square Means for breed group, sex, and season are shown in Table 3.

**Table 3:** Least Square Means by breed group, sex, and season for pre- and post-weaning traits of Ankole crossbreds

Variable^1^	Trait^2^								
	BW	W8	WWadj	WW	W18	ADG8	ADGW	ADG18	AGEW
Breed group									
AB	27.9^a^	132.2^a^	172.7^a^	167.7^b^	203.4^c^	0.435^a^	0.458^a^	0.315^b^	307.3^c^
AF	28.4^a^	126.4^ab^	173.5^a^	182.0^a^	237.7^a^	0.413^ab^	0.433^ab^	0.395^a^	353.8^a^
AJ	25.8^b^	118.4^dc^	152.8^b^	153.1^c^	218.4^bc^	0.387^bc^	0.396^c^	0.341^b^	325.7^bc^
AS	27.5^a^	122.7^bc^	156.1^b^	151.8^c^	219.2^bc^	0.398^b^	0.400^bc^	0.351^b^	310.0^c^
AJxS	27.7^a^	115.7^d^	159.3^b^	165.1^b^	226.9^ab^	0.368^c^	0.399^bc^	0.357^ab^	343.3^ab^
ASxJ	27.5^a^	123.1^bc^	159.2^b^	164.5^b^	223.5^ab^	0.398^b^	0.398^c^	0.359^ab^	345.7^a^
Sex									
F	27.0^a^	119.5^a^	161.3^a^	164.9^a^	229.5^a^	0.386^b^	0.412^a^	0.393^a^	339.3^a^
M	27.9^b^	125.9^b^	165.2^a^	164.6^a^	216.9^b^	0.410^a^	0.429^a^	0.333^b^	323.2^b^
U	27.5^a^	123.9^b^	160.3^a^	162.6^a^	218.2^b^	0.402^ab^	0.400^a^	0.332^b^	330.4^ab^
Season									
SDS	26.5^a^	121.2^b^	162.2^ab^	166.8^a^	228.2^a^	0.395^a^	0.411^a^	0.384^a^	340.8^a^
LRS	28.1^b^	126.7^a^	166.1^a^	169.9^a^	210.3^b^	0.411^a^	0.421^a^	0.314^c^	336.6^ab^
LDS	28.2^b^	123.3^ab^	163.1^ab^	162.5^ab^	220.9^a^	0.398^a^	0.418^a^	0.342^b^	325.1^ab^
SRS	27.0^a^	121.3^b^	157.7^b^	156.9^b^	226.6^a^	0.394^a^	0.406^a^	0.372^a^	321.4^b^

^1^ *AB* Ankole × Brown Swiss, *AF* Ankole × Holstein Friesian, *AJ* Ankole × Jersey, *AS* Ankole × Sahiwal, *AJxS* AJ × Sahiwal, *ASxJ* AS × Jersey, *F* female, *M* male, *U* calves with unknown sex, *SDS* short dry season (Jan–Feb), *LRS* long rainy season (Mar–May), *LDS* long dry season (Jun–Aug), *SRS* short rainy season (Sep–Dec)

^2^ *BW* birth weight (kg), *W8* weight (kg) at 8 months of age, *WW* weaning weight (kg), *W18* weight (kg) at 18 months, *ADG8* average daily gain (g) to 8 months, *ADGW* pre-weaning daily gain (g), *ADG18* post-weaning daily gain (g) up to 18 months, *AGEW* age at weaning (days). Mean values with different letters are significantly different (*P* < 0.05)

### Comparison of breed groups

The AJ calves were significantly lighter at birth (25.8 kg) than the other breed groups, which did not differ from each other (27.5–28.4 kg) (Table 3). For WWadj, AB and AF were heavier than the other four groups (AJ, AS, AJxS, and ASxJ); however, without adjustment for weaning age, AF was significantly heavier than all other breed groups. Furthermore, AB, ASxJ, and AJxS did not differ significantly from each other but were all heavier than AJ and AS. For W8, the same tendency could be seen but only AB was significantly different from the last four groups. At 18 months, AF was heavier than AJ, AS, and also AB. Although AJxS and ASxJ were approximately 14 and 11 kg lighter than AF, respectively, the difference was not significant (Table 3).

Generally, the trends for the average daily gain traits followed those for the weights; AB and AF had higher the average daily weight gain, but these differences were not always significantly different from the other four breed groups. A general trend was that the daily weight gain reached maximum at or before weaning and decreased thereafter up to 18 months. AF and AB calves performed similarly in daily weight gain up to weaning; however, at 18 months of age, AF outperformed AB significantly (Table 3).

## Discussion

### Comparison of breed groups

#### Birth weight

The BW of AF and AB crosses were not significantly different from each other; however, they were significantly higher than those of AS, AJ, AJxS, and ASxJ crosses (Table 3). The mean BW of AF observed in this study was higher than that reported with Sanga × Friesian cattle in Ghana (23.9 kg) (Apori and Hagan 2014). For the same genotype (AF) investigated in Democratic Republic of Congo, Kibwana et al. (2015) reported birth weight of 23.8 kg in non-supplemented group of animals and 24.8 kg in supplemented group. In other crossbreeding programs lighter, comparable or heavier birth weights were reported. Segura-Correa et al. 2017 reported 33.3 kg birth weight in Brown Swiss × Guzerat in Mexico, while in Ethiopia Haile et al. (2011) reported 26 kg in Friesian × Boran.

#### Weight at 8 months

The comparison of mean weight for AF at 8 months was not done because literature was not available. However, in other crossing breeding programs involving Friesian Obese et al. (2013) reported 94.2 kg in Friesian × Sanga at the age of 7 months in Ghana, while in Ethiopia Haile et al. (2011) reported 92.1 kg in Friesian × Boran at 6 months of age.

#### Weaning weight

The weaning weight (both WWadj and WW) for AF in this study was higher than the mean (148.7 kg) reported by Sottie et al. (2009) in Sanga × Friesian at the age of 365 days in Ghana and in the same station Obese et al. (2013) reported 128 kg for the same breed and age. In Gambia, Diack et al. (2004) reported 95.5 and 95.0 kg at 300 days in Friesian × N’dama and Jersey × N’dama, respectively. Segura-Correa et al. 2017 reported higher WW (178.2 kg) in Brown Swiss × Guzerat at the age of 210 days in Mexico.

#### Weight at 18 months

For body weight at 18 months in this study, AF outperformed other crossbreds. Weights of AF (238 kg) were comparable to those (248 kg) obtained on farm in Ugandan AF cross of similar age (Galukande 2010). Lower weights (159.9 kg) were reported by Obese et al. (2013) in Sanga-Friesian crossbreds. In Gambia, Diack et al. (2004) reported comparable results 246.1 and 235.8 kg in Friesian × N’dama and Jersey × N’dama, respectively; however, unlike the present study, these were body weights at 3 years of age.

#### Average daily weight gain

The daily average weight gains for AF obtained in this study were higher than those reported in Ghana, where Sottie et al. (2009) obtained 0.32 and 0.27 kg for ADG8 and ADG18, respectively, in Sanga × Friesian and in the same station Obese et al. (2013) reported 0.33 and 0.23 kg for ADG7 and ADG12, respectively. In Ethiopia, Haile et al. (2011) reported 0.511 kg for ADG6 and 0.302 kg ADG24 in Freisian × Boran.

#### General comments

In general, AB seems to do very well during the first 8 or 10 months, as well as or better than the AF cross. However, after weaning, growth decreased and the W18 was the lowest among all breed groups. By that time, the AF was the heaviest. Because the weaning age for AF was higher than for AB, the actual weight at weaning for AF was higher than all other breed groups. Because the time for weaning is decided subjectively, based on the perceived survivability, it is difficult to know if this higher weaning age for AF is truly warranted. If AF calves really need to be older (and thus heavier) when weaned, it would be interesting to find out why this would be true. This would require a more dedicated study with predetermined weaning ages and possibly also studies of grazing behavior.

Even though the AF cross was the heaviest at 18 months, the superiority compared with, say, AJ was only about 20 kg. The difference in body weight for purebred Holstein Friesian and Jersey at that age would be expected to be in the range of 130–139 kg under good conditions (e.g., Heinrichs and Hargrove 1987), which would lead to an expected difference between AF and AJ of 65–70 kg, disregarding any heterosis. The rather small differences between breed groups are probably because environmental conditions (e.g., feed, but also temperature and humidity stress) do not allow for the full expression of the genetic potential in the exotic crosses.

It would also be of great interest to follow up these breed groups after slaughter. Perhaps the difference in carcass weight would not show the same pattern as those for, say, W18. It could be hypothesized that the largest difference in W18 (between AF and AB) would be diminished or evened out, if AB has a better dressing percentage than AF. According to Bozkurt and Dogan (2016), the dressing percentage at 18 months of age was 53.6% for AB and 52.6% for AF.

## Sex

In many cattle studies, males are heavier than females at all ages (e.g., Casas et al. 2011). Also in our study, males remained significantly heavier than females up to 8 months of age; however, the relation between sexes was reversed at weaning to 18 months of age. This could be due to sale of fast-growing male calves at 6 months and older depending on the demand, while most of the female calves were kept for replacement. In studies with less severe culling of males, males tended to be heavier than females up to 18 months or 2 years; however, in one study, females (164.3 kg) were heavier than males (151.4 kg) at 18 months of age (Obese et al. 2013).

### Season

Some of the body weight and weight gain traits were affected by season of birth. Heavier calves were born in LRS and LSD, and those born in LRS tended to be heavier up to weaning. However, at 18 months, they had the lowest weight. Of course, season of birth should be viewed upon as a series of seasons following each other and this makes interpretation very difficult for such a long period as 18 months. Even for BW, it is rather the season during pregnancy than the actual season at birth that is influencing the weight. It may be that some of these animals enter into an (un)favorable combination of growth or development period and season.

### Time trends

**Fig. 1:**
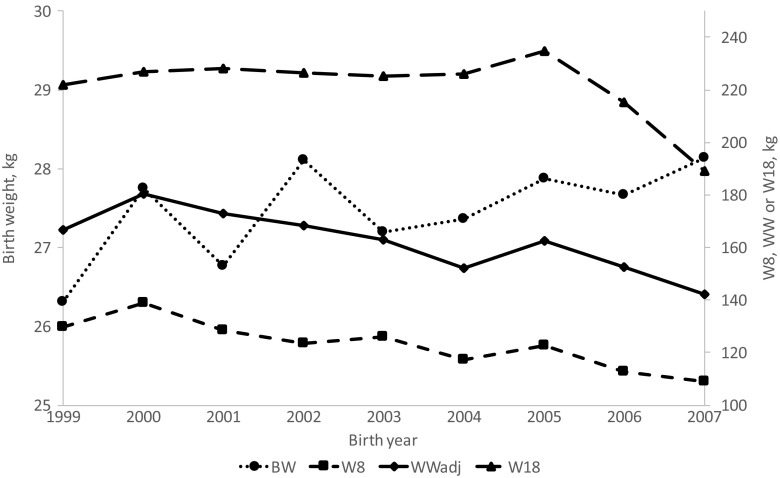
The trend of birth weight (BW), weight at 8 months (W8), age-adjusted weaning weight (WWadj), and weight at 18 months (W18) over birth years

**Fig. 2:**
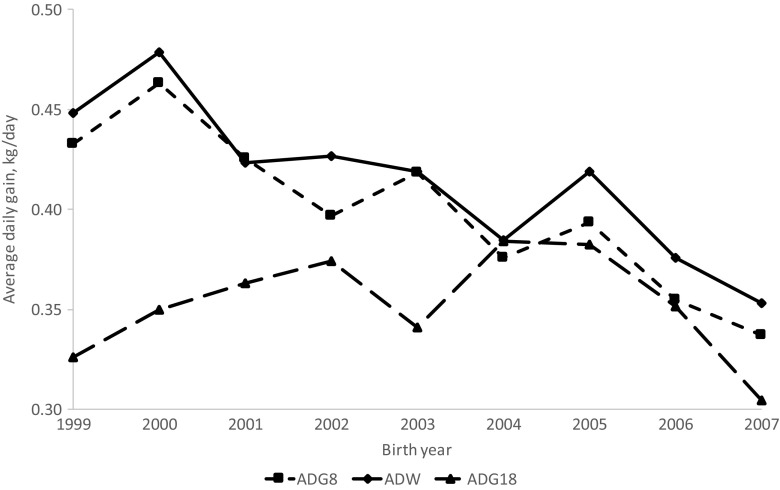
The trend of average daily gain to 8 months (ADG8), to weaning (ADGW), and to 18 months (ADG18) over birth years

There was a significant increase in BW over the years, but also a significant decrease in both W8 and WWadj as well as for corresponding growth rates (ADG8 and ADGW) (Figs. 1 and 2). Percentage-wise, this is more than 2% decrease of the live weight per year for W8 and WWadj and 3% for the growth rates. The reasons for this decline are unknown but could be related to herd variability in management practices and climatic conditions, whether this trend is particular to the Songa station is also unknown but should be a reason for careful monitoring of the future environmental conditions.

## Conclusion

The main conclusion from this study is the existence of differences in the performance of different Ankole crossbreds. Generally, AF was heavier compared to other breed groups; however, the difference in body weight was small compared to its expected genetic potential. This highlights the importance of environmental factors in contributing to the variation in pre- and post-weaning growth traits.
